# Entanglement Crossovers Under Time-Dependent U(1) Symmetry Breaking in Monitored Quantum Circuits

**DOI:** 10.3390/e28101120

**Published:** 2026-10-13

**Authors:** Terrence Lingzhu Zhao, Zhou Shangnan

**Affiliations:** 1 Department of Physics, New York University, New York, NY 10003, USA; lz3613@nyu.edu; 2 Department of Physics, Stanford University, Stanford, CA 94305, USA; 3 GreenQubit AI, 3029 Greenwood Drive, Fremont, CA 94536, USA

**Keywords:** monitored quantum circuits, measurement-induced phase transition, U(1) symmetry, entanglement dynamics, symmetry breaking, quantum information

## Abstract

Conservation laws reshape quantum information dynamics by restricting how scrambling can occur. In monitored circuits, this raises a natural dynamical question: when a symmetry constraint is gradually relaxed, how quickly do the entanglement dynamics forget the symmetric regime? We address this question in one-dimensional monitored random circuits by driving the two-qubit gate ensemble from exact U(1) conservation toward unconstrained Haar dynamics. The response is not instantaneous: the entanglement retains a finite-depth memory of the conserving dynamics and crosses over toward the unconstrained behavior over a rate-dependent time window. Operational 25%, 50%, and 75% crossover times all move to later circuit depths for slower drives. At p=0.4, the same rate ordering is also observed through L=32 in a finite-time matrix-product-state consistency check with a limited trajectory ensemble. By contrast, the weak fixed perturbations studied here, $\epsilon \left(t\right)=\epsilon_{0}\leq 0.1$, remain close to the conserving reference over the same finite observation window. Within this finite-time window, the driven crossover is therefore not reproduced merely by waiting under the fixed perturbations tested. Purification dynamics from a maximally mixed state exhibit the same qualitative response. We therefore interpret the results as a finite-time dynamical crossover from symmetry-constrained dynamics toward less constrained scrambling, rather than as evidence for a universal crossover exponent or a microscopic noise law. The study provides a controlled setting for asking how long symmetry-dependent information-processing behavior persists as a conservation law is deliberately weakened.

## 1. Introduction

Hybrid quantum circuits provide a minimal setting for studying how unitary scrambling, measurement, and conservation laws jointly control quantum information. Their dynamics combine local unitary gates, which generate and spread entanglement, with local projective measurements, which tend to reduce it. The competition between these processes can produce a measurement-induced phase transition (MIPT) [1,2,3,4]. At low measurement rates, the late-time entanglement entropy follows a volume law,

(1) S∼L,

whereas sufficiently frequent measurements produce an area-law phase,

(2) S∼O(1).

A critical measurement rate $p_{c}$ separates these two regimes.

Conservation laws introduce additional structure into this competition. In a U(1)-symmetric circuit, the conserved charge spreads diffusively even though generic operator components spread ballistically [5,6]. The symmetry restricts the allowed gates and can reduce their entanglement-generating ability. In the circuit protocol studied here, this restriction is associated with a lower finite-size transition location than in the corresponding unconstrained circuit [7]. This distinction motivates a dynamical response question: how rapidly do monitored entanglement dynamics change when the local gate ensemble is deliberately driven away from exact U(1) conservation?

We address this question with an externally prescribed, nonstationary circuit protocol. Each two-qubit gate is obtained by interpolating between a U(1)-conserving gate and an unconstrained Haar-random gate, followed by a polar-decomposition projection back onto the unitary group. A control parameter ϵ(t) specifies the instantaneous gate ensemble. We consider linear and exponential profiles,

(3) $$\begin{array}{cc} \epsilon_{\mathrm{lin}}\left(t\right) & =\min(1,\gamma t), \end{array}$$

(4) $$\begin{array}{cc} \epsilon_{\exp}\left(t\right) & =1-e^{-\gamma t}, \end{array}$$

where γ controls the rate of the imposed drive. The linear protocol reaches the unconstrained endpoint at t=1/γ, whereas the exponential profile approaches it asymptotically. These functions are chosen as simple controlled paths between the same two gate ensembles; ϵ(t) is not an accumulated error probability, a charge-decay law, or a microscopic leakage rate.

Because the protocols themselves fix the direction of the interpolation, the nontrivial numerical information lies in the finite-time response. We therefore ask when the entropy departs from the conserving reference, how broad the crossover is, and how the response times depend on the driving rate. Our numerical results establish five main findings.

(i) **Distinct finite-size reference behaviors.** Tripartite-mutual-information crossings for the accessible equal-quarter sizes L=8,12,16 occur near p≃0.21 for the U(1)-conserving circuit and p≃0.34 for the unconstrained Haar circuit. Residual crossing drift prevents these values from being interpreted as asymptotically controlled thermodynamic critical points; they serve here as finite-size reference estimates.
(ii) **Rate-dependent finite-time response.** For every nonzero ramp rate studied, the entropy departs from the conserving reference and moves toward the unconstrained reference. For the linear ramp, we characterize this response with the operational threshold times $t_{25}$, $t_{50}$, and $t_{75}$. All three thresholds move to later circuit depths as γ decreases, so the rate dependence does not rely on privileging a single midpoint. The interval $t_{75}-t_{25}$ additionally quantifies the temporal width of the crossover.
(iii) **Larger-system MPS validation.** An independent MPS implementation reproduces the exact-statevector dynamics at L=16 in a controlled weak-entanglement benchmark and remains converged in bond dimension through L=32 at p=0.4. For L=16, 24, 32, the slower linear ramp (γ=0.02) has a later midpoint and 75% threshold than the faster ramp (γ=0.05). The thresholds are not monotonic in *L*, so these data serve as a larger-system consistency check rather than a thermodynamic extrapolation.
(iv) **Static weak-symmetry-breaking control.** To distinguish the driven crossover from cumulative evolution under a weak fixed perturbation, we also simulate $\epsilon \left(t\right)=\epsilon_{0}$ with $\epsilon_{0}\in \left\{0.01,0.02,0.05,0.10\right\}$ at L=12, p=0.1,0.2, and T=200. These static curves remain close to the conserving reference over the simulated window and show no sustained motion toward the Haar reference. This finite-time control does not determine the asymptotic relevance of arbitrarily weak symmetry breaking, but it demonstrates that the observed ramp response is not reproduced merely by waiting under a weak fixed gate ensemble.
(v) **Crossover in purification dynamics.** Purification simulations initialized from the maximally mixed state exhibit the same qualitative response as the pure-state simulations. Within the finite sizes, ramp rates, and evolution times studied, this agreement shows that the observed crossover is not specific to entanglement growth from the Néel initial state.

The scope of these statements is deliberately finite-time and protocol specific. The static-ϵ control constrains the response over T=200 but does not determine the asymptotic fate of an arbitrarily weak symmetry-breaking perturbation. We also do not identify γ or ϵ with a physical decoherence or leakage rate. Actual device noise is non-unitary and requires explicit quantum channels or multilevel models. We therefore treat possible NISQ and quantum-machine-learning connections as outlooks rather than conclusions tested by the present simulations.

The remainder of the paper is organized as follows. Section 2 reviews entanglement scaling, MIPTs, the distinction between our coherent gate interpolation and physical leakage, and the purification picture. Section 3 defines the U(1)-conserving and Haar reference circuits and extracts their finite-size crossing estimates. Section 4 introduces the two driven protocols and presents the entanglement and purification results. Section 5 summarizes what the simulations establish, states their finite-size and modeling limitations, and discusses possible extensions to hardware noise and trainable quantum circuits. Section 6 concludes.

## 2. Background

Before presenting our model, we collect the background material that the rest of the paper builds on: the classification of entanglement scaling into area and volume laws (Section 2.1), the phenomenology of measurement-induced phase transitions (Section 2.2), the physics of leakage in superconducting transmon qubits (Section 2.3), and the purification picture that connects mixed-state and pure-state dynamics (Section 2.4).

### 2.1. Entanglement Scaling: Area and Volume Laws

We begin by reviewing the two ways entanglement can scale in quantum many-body systems: the volume law and the area law. The measurement-induced transition runs between these two regimes: the former occurs for measurement rates *p* smaller than a critical rate $p_{c}$, and the latter occurs for *p* greater than $p_{c}$.

To formalize the scaling of entanglement, we consider a lattice system Λ consisting of *N* sites, with the total Hilbert space given by the tensor product of local finite-dimensional Hilbert spaces, $H={⨂}_{i\in \Lambda }H_{i}$. We divide the system into a spatial subsystem *A* and its complement B=Λ∖A.

For any pure state |Ψ〉∈H, the reduced density matrix of subsystem *A* is obtained by tracing out the degrees of freedom in *B*:

(5) $$\rho_{A}={\mathrm{Tr}}_{B}\;(|\Psi \rangle \langle \Psi |).$$

The bipartite entanglement between *A* and *B* is quantified by the von Neumann entropy of the reduced density matrix:

(6) $$S_{A}=-\mathrm{Tr}\;\left(\rho_{A}\log\rho_{A}\right).$$

The asymptotic scaling of $S_{A}$ with respect to the characteristic size of the subregion *A* (denoted by |A|) provides a fundamental classification of quantum phases and non-equilibrium dynamics.

#### 2.1.1. The Volume Law

A quantum state satisfies a volume law if its entanglement entropy scales extensively with the number of degrees of freedom in the subsystem:

(7) $$S_{A}∼\left|A\right|.$$

In a *d*-dimensional system where the subregion *A* has a linear dimension $L_{A}$, the volume law dictates that $S_{A}\propto L_{A}^{d}$. Physically, volume-law entanglement is a hallmark of highly excited eigenstates in chaotic many-body systems that satisfy the Eigenstate Thermalization Hypothesis (ETH). In such systems, the rest of the system acts as a thermal bath for *A*, and the entanglement entropy coincides with the thermodynamic entropy. In the context of unitary dynamics, scrambling and chaotic evolution generically drive an initially unentangled state toward a volume-law phase, reflecting the delocalization of quantum information across the entire system.

#### 2.1.2. The Area Law

Conversely, a quantum state satisfies an area law if its entanglement entropy scales strictly with the size of the boundary ∂A separating subregion *A* from *B*, rather than with the bulk volume:

(8) $$S_{A}=O\left(|\partial A|\right).$$

For a *d*-dimensional system, this implies $S_{A}\propto L_{A}^{d-1}$. In the specific case of one-dimensional (1D) systems, the boundary consists of merely two points (or a constant number of cuts), leading to a saturation of the entanglement entropy: $S_{A}=O\left(1\right)$, independent of the subsystem size |A| as |A|→∞. The area law represents a non-trivial restriction on the accessible Hilbert space. It is rigorously proven for the ground states of gapped, local Hamiltonians in 1D and is widely observed in higher dimensions. The physical intuition behind the area law is that quantum correlations in these low-energy states are mediated by strictly local interactions and decay exponentially with distance. Consequently, the entanglement between *A* and *B* is dominated by the localized degrees of freedom situated along the boundary ∂A.

#### 2.1.3. Computation from the Schmidt Decomposition

For a bipartition of the chain into *A* and *B*, any pure state admits a Schmidt decomposition

(9) $$|\Psi \rangle =\sum_{\alpha =1}^{\chi_{\max}}\lambda_{\alpha }\;|\psi_{\alpha }^{A}\rangle \;|\psi_{\alpha }^{B}\rangle ,\;\sum_{\alpha =1}^{\chi_{\max}}\lambda_{\alpha }^{2}=1,$$

with Schmidt coefficients $\lambda_{\alpha }\geq 0$, where $\chi_{\max}=\min\left(\dim H_{A},\dim H_{B}\right)$. For a chain of *L* qubits cut at the centre, $\chi_{\max}=2^{L/2}$. The eigenvalues of the reduced density matrix $\rho_{A}={\mathrm{Tr}}_{B}\left|\Psi \rangle \langle \Psi \right|$ are the squared Schmidt coefficients $\lambda_{\alpha }^{2}$, so the entanglement (von Neumann) entropy is

(10) $$S=-\sum_{\alpha =1}^{\chi_{\max}}\lambda_{\alpha }^{2}\log\;\left(\lambda_{\alpha }^{2}\right).$$

This is how all entropies reported below are computed; the simulations store the full wavefunction, so the sum runs over the complete Schmidt spectrum.

### 2.2. Measurement-Induced Phase Transitions

In hybrid quantum circuits—where unitary gates compete with projective measurements—a new class of non-equilibrium phase transitions has been discovered: the measurement-induced phase transition (MIPT) [1,8,9,10,11]. We work throughout in one spatial dimension, so that a subsystem *A* is an interval with volume |A|∼L and a boundary ∂A consisting of a fixed number of points, |∂A|=O(1). At low measurement rates, unitary scrambling dominates and the steady-state entanglement entropy obeys a volume law,

(11) $$S_{A}\propto \left|A\right|∼L,$$

whereas at high measurement rates, frequent measurements collapse quantum superpositions and disentangle the system, driving the entropy to an area law,

(12) $$S_{A}\propto \left|\partial A\right|=O\left(1\right).$$

A sharp critical point $p_{c}$ separates these two phases; at $p_{c}$ the entanglement grows logarithmically, $S_{A}∼\log L$, interpolating between the two regimes.

The dichotomy between area and volume laws frames the underlying mechanism of the MIPT explored in this work. While area laws traditionally describe static, low-energy ground states, MIPTs demonstrate that non-unitary dynamics can dynamically arrest chaotic volume-law growth. Repeated projective measurements continually extract information and collapse quantum superpositions, effectively truncating long-range correlations and localizing entanglement. The competition between unitary scrambling (driving $S_{A}\propto \left|A\right|$) and measurement-induced collapse drives the dynamical transition into an area-law phase ($S_{A}\propto \left|\partial A\right|$), mimicking the localized entanglement structure of a ground state despite the high-energy, non-equilibrium nature of the repeated circuit dynamics.

### 2.3. Physical Leakage and Open-System Noise

Many quantum devices are designed around an effective two-level computational subspace, while the physical hardware is open and may contain additional levels. In a transmon, for example, population can leave {|0〉,|1〉} and enter higher levels such as |2〉; this process is called leakage [12,13,14,15]. Amplitude damping, dephasing, and leakage are described by non-unitary quantum channels (or by an enlarged-system unitary description), and leakage additionally changes the relevant local Hilbert space.

These physical processes are conceptually distinct from the model studied here. Our interpolating two-qubit gates remain exactly unitary within the qubit Hilbert space, and the control parameter ϵ(t) specifies the gate ensemble at each circuit depth. We therefore do not interpret ϵ(t) as a leakage probability or identify γ with a device noise rate. The comparison with hardware is limited to the broader question of how symmetry-sensitive dynamics respond when symmetry-breaking effects become more important with circuit depth. A quantitative hardware model would require explicit open-system channels or a multilevel transmon description, which we leave for future work.

### 2.4. Purification and the Ancilla Picture

To understand why the dynamics of a mixed state are similar to those of a pure state, we employ the ancilla picture. A mixed density matrix $\rho_{S}$ of the system *S* can always be purified by introducing a fictitious reference system *R* that is entangled with *S*.

Let the system *S* consist of *L* qubits. We introduce a reference system *R*, which also consists of *L* qubits. The initial maximally mixed state of the system, $\rho_{S}\left(0\right)\propto I_{2^{L}}$, can be represented as the reduced density matrix of a pure state $|\Psi_{SR}\rangle$ in the enlarged Hilbert space $H_{S}⊗H_{R}$:

(13) $$|\Psi_{SR}\left(0\right)\rangle =\overset{L}{\underset{i=1}{⨂}}\frac{1}{\sqrt{2}}\left({|0\rangle }_{S_{i}}{|0\rangle }_{R_{i}}+{|1\rangle }_{S_{i}}{|1\rangle }_{R_{i}}\right).$$

In this state, every qubit in *S* is maximally entangled with a corresponding qubit in *R*. The von Neumann entropy of system *S*, $S(\rho_{S})$, is exactly the entanglement entropy between *S* and *R*. During the time evolution, the unitary gates and measurements act only on the system *S*, whereas the reference system *R* evolves under the identity operation. For a fixed measurement trajectory m, let $K_{m}\left(t\right)$ denote the ordered product of all unitary gates and measurement projectors up to time *t*. The normalized joint state is

(14) $$|\Psi_{SR}^{\left(m\right)}\left(t\right)\rangle =\frac{\left(K_{m}\left(t\right)⊗I_{R}\right)|\Psi_{SR}\left(0\right)\rangle }{∥\left(K_{m}\left(t\right)⊗I_{R}\right)|\Psi_{SR}\left(0\right)\rangle ∥}.$$

Conditioned on the measurement outcomes, this normalized joint state remains pure even though $K_{m}\left(t\right)$ is not unitary. The purification of the mixed state corresponds to the decoupling of *S* from *R*. The entropy $S(\rho_{S})$ measures how much entanglement remains between the system *S* and the reference *R*. For sparse measurements, $p<p_{c}$, unitary scrambling prevents measurements from rapidly extracting the encoded quantum information. The system therefore remains entangled with the reference, and $S(\rho_{S})$ stays nonzero over times that grow rapidly with system size. This regime is conventionally called the mixed phase. For frequent measurements, $p>p_{c}$, information is extracted more rapidly, causing the system to decouple from the reference. Consequently, $S(\rho_{S})$ approaches zero and the conditioned state of *S* becomes pure; this regime is called the pure or purifying phase. The purification transition therefore provides a system–reference diagnostic closely related to the measurement-induced entanglement transition.

## 3. U(1)-Conserving and Unconstrained Circuits

### 3.1. The U(1)-Conserving Hybrid Circuit

In this section we consider a one-dimensional chain of *L* spin-1/2 qubits with a U(1) conservation law (conserved total $S^{z}$). The primary numerical data in this work are obtained from exact statevector simulations: the full $2^{L}$-dimensional wavefunction is stored and evolved without truncation, so the dynamics, measurement statistics, and entanglement readout carry no truncation error at the accessible sizes (L≤16). To probe larger systems in a controlled weak-entanglement regime, we additionally implement a matrix-product-state (MPS) version of the same circuit; its validation and convergence tests are presented in Section 4.3.3.

The numerical simulation and analysis workflow was developed and executed using Anthropic’s Fable 5. The authors reviewed and validated the code and numerical outputs and take full responsibility for the reported results. The supplied reproduction environment is pinned to Python 3.14.7, NumPy 2.5.3, SciPy 1.18.1, pandas 3.0.5, and Matplotlib 3.11.1, the latest stable releases checked on 9 September 2026.

**Figure 1:**
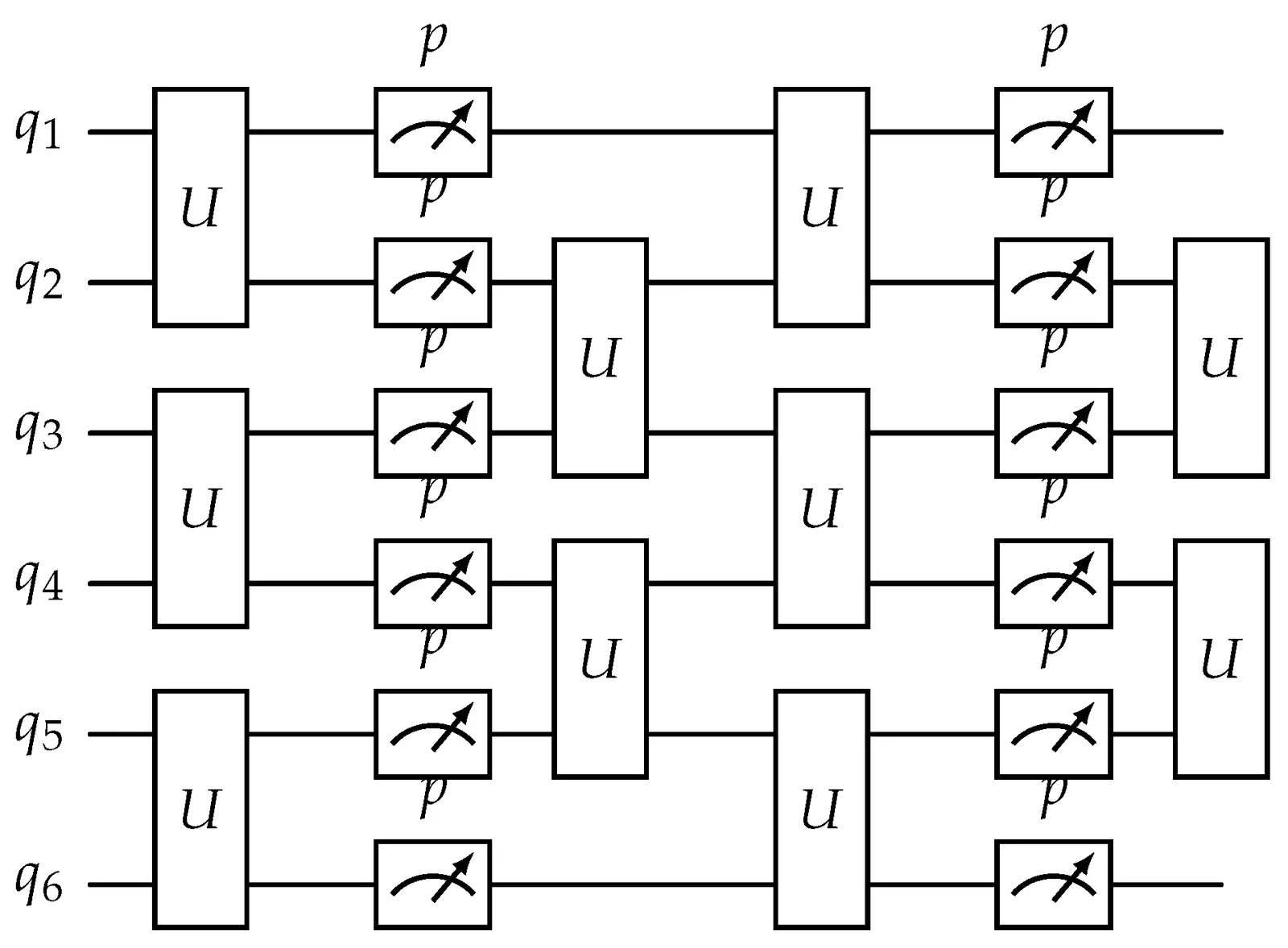
Two time steps of the hybrid quantum circuit. Two-site unitaries *U* are applied in a staggered (brickwork) pattern. Sites are labeled $q_{1},\ldots ,q_{L}$: the first unitary layer acts on the bonds $\left(q_{i},q_{i+1}\right)$ with *i* odd, followed by a projective measurement layer acting on each site with probability *p*, followed by a unitary layer on the bonds with *i* even.

As in Refs. [5,16], the time evolution is governed by a random circuit of unitary gates and projective measurements. We use a discrete-time circuit with a staggered arrangement of two-site unitaries (Figure 1). Each time step *t* is decomposed into three layers. Labeling the sites $q_{1},\ldots ,q_{L}$ and the bond between $q_{i}$ and $q_{i+1}$ by its left site index *i*, these are: a layer of two-site unitaries on the odd bonds i=1,3,5,… ($U_{\mathrm{odd}}$), a stochastic measurement layer (M), and a layer of unitaries on the even bonds i=2,4,6,… ($U_{\mathrm{even}}$). Within each unitary layer, the gates act on disjoint pairs of sites and therefore commute, so

(15) $$U_{\mathrm{odd}}\left(t\right)=\prod_{i\;\mathrm{odd}}U_{i,i+1}\left(t\right),\;U_{\mathrm{even}}\left(t\right)=\prod_{i\;\mathrm{even}}U_{i,i+1}\left(t\right)$$

are unambiguous.

The measurement layer acts between the two unitary layers and cannot be commuted through them, so the two unitary layers are never applied consecutively and do not combine into a single step unitary. Each site is measured independently with probability *p*; writing $S_{t}$ for the randomly drawn subset of measured sites and $m_{i}\in \left\{↑,↓\right\}$ for the outcome at site *i*, the (non-unitary) measurement operator of the layer is the product of single-site projectors

(16) $$M_{m}\left(t\right)=\underset{i\in S_{t}}{⨂}P_{m_{i},\;i},\;P_{↑}=|↑\rangle \langle ↑|,\;P_{↓}=|↓\rangle \langle ↓|.$$

One time step therefore maps

(17) $$|\Psi \left(t+1\right)\rangle =\frac{U_{\mathrm{even}}\left(t\right)\;M_{m}\left(t\right)\;U_{\mathrm{odd}}\left(t\right)\;|\Psi \left(t\right)\rangle }{∥\;M_{m}\left(t\right)\;U_{\mathrm{odd}}\left(t\right)\;|\Psi \left(t\right)\rangle \;∥},$$

where operators act from right to left, and the outcome string m is drawn with the Born probability $P\left(m\right)=∥M_{m}\left(t\right)\;U_{\mathrm{odd}}{\left(t\right)\;|\Psi \left(t\right)\rangle ∥}^{2}$ (the final unitary layer does not affect the norm). Each realization of the measurement locations and outcomes defines a quantum trajectory, and all reported observables are averaged over trajectories.

Two commutation properties of these projectors are worth making explicit. First, projectors acting on distinct sites commute with one another, so the outcome distribution of a measurement layer is independent of the order in which the sites within that layer are measured. Second, and more importantly, $P_{↑}$ and $P_{↓}$ are diagonal in the $S^{z}$ basis and therefore commute with the total charge,

(18) $$\left[P_{↑/↓,\;i},\;S_{\mathrm{tot}}^{z}\right]=0\;\mathrm{for}\;\mathrm{all}\;i.$$

The measurements are thus performed in the charge basis and do not themselves break the U(1) symmetry: being diagonal in the $S^{z}$ basis, they neither create nor transfer weight between charge sectors. At γ=0 the total charge is therefore conserved by the full monitored dynamics—unitary gates and measurements alike—and the symmetry breaking introduced in Section 4 enters exclusively through the gates via ϵ(t). The projectors do not, however, commute with the two-site gates: $P_{↑}$ on a single site distinguishes |↑↓〉 from |↓↑〉, which the block $U_{Q=0}$ mixes. It is precisely this non-commutation that generates the competition between scrambling and measurement-induced disentanglement.

**Figure 2:**
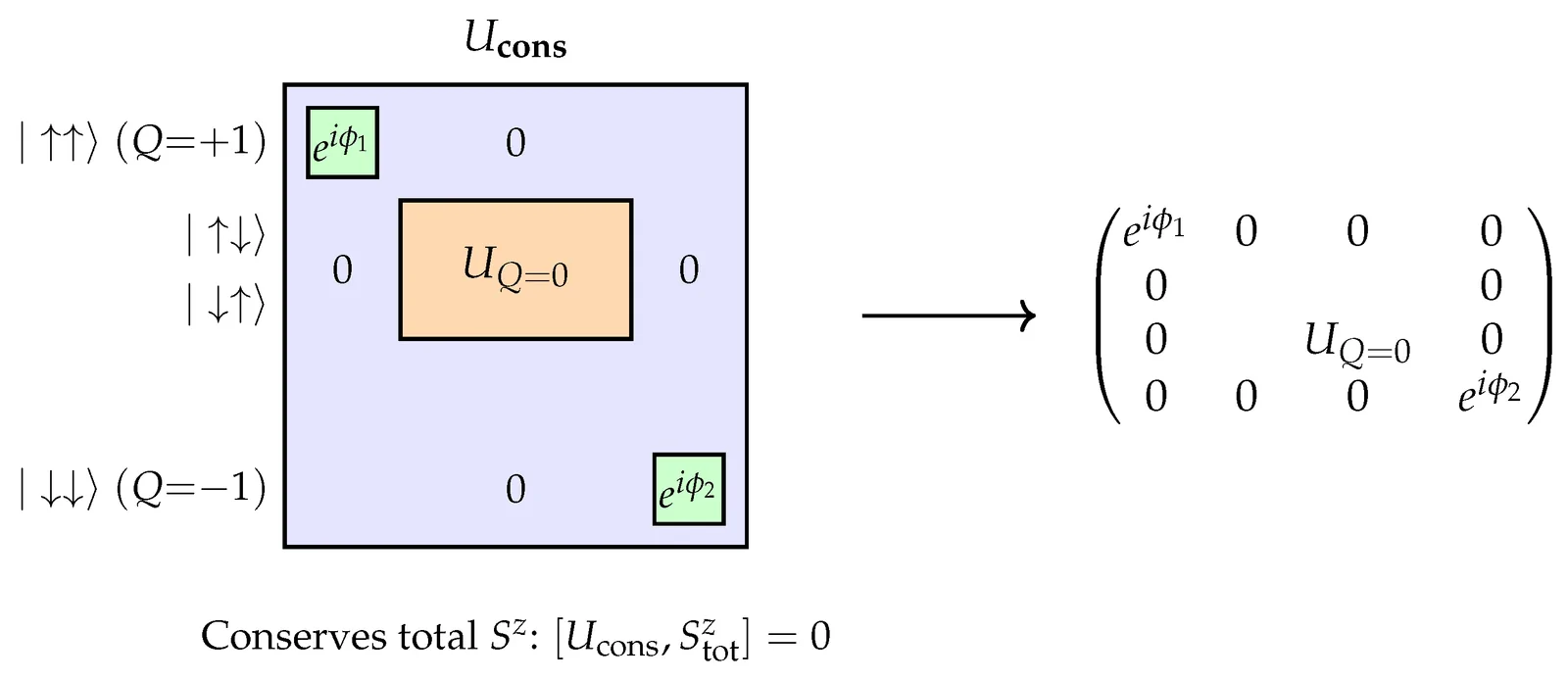
Block-diagonal structure of the U(1)-conserving two-site unitary gate. The 2×2 block $U_{Q=0}$ acts on the $S_{\mathrm{tot}}^{z}=0$ subspace (|↑↓〉, |↓↑〉), while the polarized states |↑↑〉 ($S_{\mathrm{tot}}^{z}=+1$) and |↓↓〉 ($S_{\mathrm{tot}}^{z}=-1$) acquire only the phases $e^{i\phi_{1}}$ and $e^{i\phi_{2}}$.

The explicit U(1)-conserving unitary gate, acting on the basis {|↑↑〉,|↑↓〉,|↓↑〉,|↓↓〉}, is block diagonal in the total magnetization (Figure 2),

(19) $$U_{\mathrm{cons}}=\left(\begin{array}{ccc} e^{i\phi_{1}} & \begin{array}{cc} 0 & 0 \end{array} & 0\\ \begin{array}{c} 0\\ 0 \end{array} & U_{Q=0} & \begin{array}{c} 0\\ 0 \end{array}\\ 0 & \begin{array}{cc} 0 & 0 \end{array} & e^{i\phi_{2}} \end{array}\right),\;\left[U_{\mathrm{cons}},S_{\mathrm{tot}}^{z}\right]=0.$$

Here the 2×2 central block $U_{Q=0}$ mixes |↑↓〉 and |↓↑〉 (total magnetization $S_{\mathrm{tot}}^{z}=0$) and is the dominant source of transport and operator spreading. The fully polarized states |↑↑〉 (Q=+1) and |↓↓〉 (Q=−1) acquire only the phases $e^{i\phi_{1}}$ and $e^{i\phi_{2}}$; these phases are themselves entangling unless they factorize into single-site rotations.

### 3.2. The Haar Reference Circuit and the Interpolating Gate Family

We compare the U(1)-conserving circuit throughout with an unconstrained reference circuit. In this reference circuit, every $U_{\mathrm{cons}}$ is replaced by a gate drawn from the Haar measure on U(4), the group of all two-qubit unitaries. The brickwork geometry, measurement layer, and all other parameters are kept identical. The Haar measure is the uniform distribution on that group. This circuit possesses no conservation law and realizes the generic measurement-induced transition studied in Refs. [1,17]; we refer to it as the Haar circuit.

The two circuits are the endpoints of a one-parameter family of gate ensembles, which we define here for a fixed mixing parameter; in Section 4 the same construction is used with the mixing parameter made time-dependent. For a mixing parameter ϵ∈[0,1], each application of a two-site gate draws one gate $U_{\mathrm{cons}}$ from the U(1)-conserving ensemble and one gate $U_{\mathrm{Haar}}$ from the Haar measure on U(4), forms the mixture

(20) $$M_{i}=\left(1-\epsilon \right)\;U_{\mathrm{cons}}+\epsilon \;U_{\mathrm{Haar}},$$

and applies the unitary obtained by projecting $M_{i}$ back onto the unitary group via the polar decomposition,

(21) $$U_{i}\left(\epsilon \right)=M_{i}\;{\left[M_{i}^{†}M_{i}\right]}^{-1/2},$$

i.e., the unitary factor of $M_{i}$, which is the unitary closest to $M_{i}$ in Frobenius norm. Each applied gate is therefore exactly unitary, and the circuit defines a well-posed trajectory ensemble for every ϵ. The mixing parameter controls the strength of symmetry breaking per gate. At ϵ=0, every gate is exactly U(1)-conserving and the circuit is the one defined in Section 3.1. At ϵ=1, every gate is Haar-random and the circuit is the Haar reference circuit. At intermediate ϵ, each gate violates the conservation law weakly but with certainty (an alternative stochastic construction—applying $U_{\mathrm{cons}}$ with probability 1−ϵ and $U_{\mathrm{Haar}}$ with probability ϵ—shares the same two endpoints; we have checked that both constructions exhibit the same long-time behavior). Section 3.5 studies the two endpoints ϵ≡0 and ϵ≡1; Section 4 makes ϵ time-dependent.

### 3.3. Measurement-Free Limit (p=0): Charge Hydrodynamics and Ballistic Spreading

At p=0 the circuit contains no measurements, and the dynamics reduces to a U(1)-conserving random unitary circuit of the kind studied in Refs. [6,18]. We state the resulting picture first and then briefly review how it arises.

An initially local operator develops two components that spread very differently. The non-conserved component spreads ballistically, with its front advancing at the butterfly velocity $v_{B}$. The conserved component carries the U(1) charge, spreads only diffusively, and remains concentrated near the origin. In the Haar circuit, the conserved component is absent and only the ballistic front remains. This difference provides a microscopic mechanism consistent with the lower finite-size transition location of the conserving reference discussed in Section 3.5. A reader familiar with operator hydrodynamics may proceed directly to Section 3.4.

To state this precisely, we expand a Heisenberg operator in the Pauli-string basis, $O\left(t\right)=\sum_{S}a_{S}\left(t\right)\;S$. Unitarity fixes the total weight,

(22) $$\sum_{S}{\left|a_{S}\left(t\right)\right|}^{2}=2^{-L}\;\mathrm{Tr}\;\left[O{\left(t\right)}^{†}O\left(t\right)\right]=1,$$

so the dynamics can only redistribute weight among strings, never create or destroy it. Resolving this weight in space gives the operator-weight density

(23) $$\rho \left(x,t\right)\equiv \sum_{S\in {\mathrm{basis}}_{x}}{\left|a_{S}\left(t\right)\right|}^{2}=\rho_{\mathrm{cons}}\left(x,t\right)+\rho_{\mathrm{non}-\mathrm{cons}}\left(x,t\right),$$

where ${\mathrm{basis}}_{x}$ denotes the strings whose right endpoint lies at site *x*. We have split it into the part overlapping the local U(1) charge density, $\rho_{\mathrm{cons}}$, and the remainder, $\rho_{\mathrm{non}-\mathrm{cons}}$.

These two parts behave very differently. The conserved part is constrained by charge conservation. Writing

(24) $$c\left(x,t\right)=2^{-L}\;\mathrm{Tr}\;\left[\sigma_{x}^{z}\;O\left(t\right)\right]$$

for the amplitude of O(t) on the local charge density $\sigma_{x}^{z}$, the total overlap $\sum_{x}c\left(x,t\right)$ is fixed by $[U_{\mathrm{cons}},S_{\mathrm{tot}}^{z}]=0$, so this amplitude can only be redistributed in space. In a chaotic circuit that redistribution is diffusive,

(25) $$\partial_{t}\;c\left(x,t\right)=D\;\partial_{x}^{2}\;c\left(x,t\right),$$

with diffusion constant *D*: the profile broadens as $\langle x^{2}\rangle ∼Dt$ while its integral stays fixed. The squared weight $\rho_{\mathrm{cons}}$ is not itself conserved—it is steadily converted into non-conserved strings, decaying as $t^{-1/2}$ in one dimension, which is the dissipative mechanism identified in Ref. [18]. The non-conserved part is subject to no such constraint and spreads ballistically at $v_{B}$, giving the fast front and slow diffusive core described above.

In the Haar circuit the same expansion applies, but no local operator overlaps a conserved density, so $\rho_{\mathrm{cons}}\equiv 0$ in Equation (23) and all weight is carried by the ballistic component: the profile is a front moving at $v_{B}$ with no slowly decaying core left behind, and correlations relax exponentially rather than as a power law in time [5,19]. This contrast provides a physical mechanism consistent with the reference-circuit difference measured in Section 3.5: gates constrained to transport charge diffusively can generate entanglement less efficiently than unconstrained ones.

### 3.4. Measurement-Driven Regime (p>0): Observables and Simulation Protocol

We now return to the general monitored case p>0, which is the setting for all numerical results that follow. Along a measurement trajectory, the evolution is no longer unitary and the argument leading to Equation (25) no longer applies. We expect, on general grounds, that the collapse of superpositions damps the operator’s overlap with the local charge density, and we model this phenomenologically by adding a loss term,

(26) $$\partial_{t}\;c\left(x,t\right)=D\;\partial_{x}^{2}\;c\left(x,t\right)\;-\;\Gamma \left(p\right)\;c\left(x,t\right),\;\Gamma \left(0\right)=0,\;\frac{d\Gamma }{dp}>0,$$

so that $\sum_{x}c\left(x,t\right)$, constant at p=0, decays at a rate set by the measurement rate. We emphasize that this is a decay of the operator’s charge-density correlations along a trajectory, not a loss of charge itself: as shown in Section 3.1, the projectors commute with $S_{\mathrm{tot}}^{z}$, so the total charge remains conserved on every trajectory. Equation (26) is a heuristic used only to motivate the expectation that competition between diffusive spreading and measurement-induced damping can shift the finite-size transition region of the U(1) circuit relative to the unconstrained one [7]. We do not determine Γ(p); the comparison is instead settled numerically in Section 3.5 (we note that U(1) monitored circuits also host a distinct charge-sharpening transition, separate from the entanglement transition; we focus on the latter here).

The bipartite entanglement entropy across the central cut is $S\left(L/2\right)=-\mathrm{Tr}\left(\rho_{A}\log\rho_{A}\right)$. It is obtained from the Schmidt decomposition of the full wavefunction at the central cut, Equations (9) and (10).

Unless stated otherwise, we simulate chains of L=8–16 qubits initialized in the Néel state |↑↓↑↓…〉. We evolve the circuits for T=100 layers, or T=200 for the symmetry-breaking-ramp studies, and average observables over the final 20 layers and over 60–300 independent measurement trajectories. Quoted uncertainties are standard errors of the mean, and random seeds are fixed per data set for reproducibility. To obtain a cleaner finite-size transition diagnostic we use the tripartite mutual information [3,17] $I_{3}=S_{A}+S_{B}+S_{C}-S_{AB}-S_{AC}-S_{BC}+S_{ABC}$ for contiguous quarters *A*, *B*, *C*, with the remaining quarter denoted *R* (the symbol *D* is reserved for the diffusion constant). Near the transition, $I_{3}$ is approximately scale invariant and its crossings are less affected by the strong size dependence of the raw entropy, although finite-size drift remains. We compare only equal-quarter sizes L=8,12,16, since sizes sharing ⌊L/4⌋ yield nearly identical $I_{3}$. A small systematic offset between chains with L/2 even and odd, originating from the brickwork structure, is visible in the raw entropy curves; crossings are never taken across this parity.

### 3.5. Numerical Results for the Two Reference Circuits

We compare the U(1)-conserving and unconstrained Haar circuits using two complementary quantities. The late-time half-chain entropy shows how the entanglement changes with measurement rate and distinguishes the volume-law and area-law regimes. The tripartite mutual information provides a clearer finite-size estimate of the critical measurement rate separating these regimes.

#### 3.5.1. Half-Chain Entropy

We first measure the von Neumann entropy across the central cut of the chain. Dividing the system into two equal halves, *A* and $\bar{A}$, we define

(27) $$\rho_{A}={\mathrm{Tr}}_{\bar{A}}\left[|\psi \rangle \langle \psi |\right]$$

and

(28) $$S_{1/2}=-\mathrm{Tr}\left(\rho_{A}\ln\rho_{A}\right).$$

This entropy measures the entanglement between the two halves of the system. For each measurement rate and system size, we average it over measurement trajectories and over the final 20 circuit layers.

The dependence of $S_{1/2}$ on system size distinguishes the two measurement-induced phases. In the volume-law phase,

(29) $$S_{1/2}\propto L,$$

so the entropy increases as the system becomes larger. In the area-law phase,

(30) $$S_{1/2}=O\left(1\right),$$

so it becomes approximately independent of system size. The half-chain entropy therefore shows how increasing the measurement rate suppresses entanglement, although it does not by itself give a precise finite-size estimate of the critical point.

**Figure 3:**
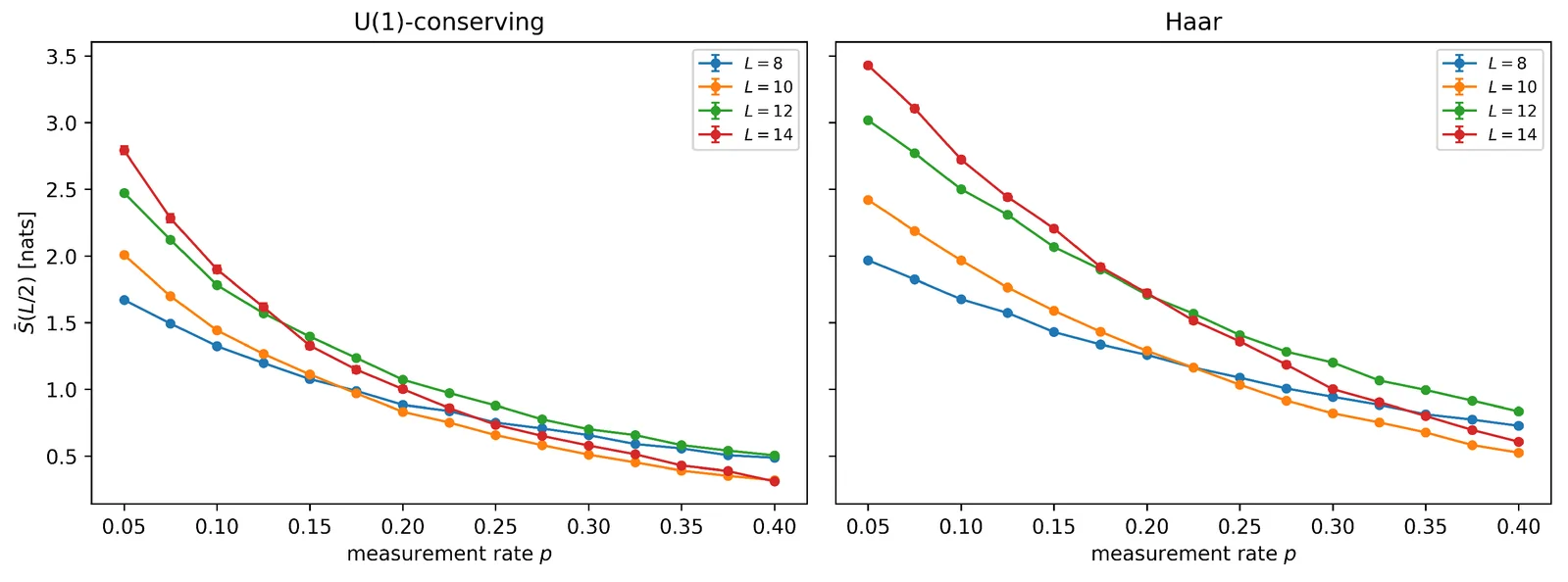
Late-time half-chain entropy $S_{1/2}$ versus measurement rate *p* for system sizes L=8, 10, 12, 14. Results are shown for the U(1)-conserving circuit (**left**) and the unconstrained Haar circuit (**right**) under the same measurement protocol. The entropy is averaged over the final 20 circuit layers and over measurement trajectories. Error bars denote the standard error of the mean.

Figure 3 shows that the half-chain entropy decreases as the measurement rate increases in both circuits. At small *p*, the entropy increases noticeably with *L*, indicating volume-law entanglement. At large *p*, the curves for different system sizes approach one another, indicating area-law entanglement.

At every simulated measurement rate, the conserving circuit has less half-chain entanglement than the Haar circuit. The U(1) constraint restricts the allowed two-qubit gates and reduces their entanglement-generating ability relative to unconstrained Haar-random gates [6,7]. As a consistency check, all measured entropies remain below the corresponding Page value, which is the mean half-chain entropy of a Haar-random pure state.

#### 3.5.2. Tripartite Mutual Information and Finite-Size Crossing Estimates

To locate the two reference transition regions more cleanly, we calculate the tripartite mutual information $I_{3}$. We divide the periodic chain into four contiguous regions of equal size, denoted by *A*, *B*, *C*, and *R*, and define

(31) $$I_{3}\left(A:B:C\right)=S_{A}+S_{B}+S_{C}-S_{AB}-S_{AC}-S_{BC}+S_{ABC}.$$

Here, $S_{X}$ is the von Neumann entropy of region *X*.

Near a measurement-induced transition, $I_{3}$ is approximately scale-invariant, so curves from different sizes develop crossings near the transition region. For the exact statevector sizes accessible here, these crossings are useful finite-size diagnostics but do not by themselves provide a controlled thermodynamic-limit extrapolation. Because the construction requires four equal regions, we use L=8,12, and 16 and compare only these equal-quarter sizes.

**Figure 4:**
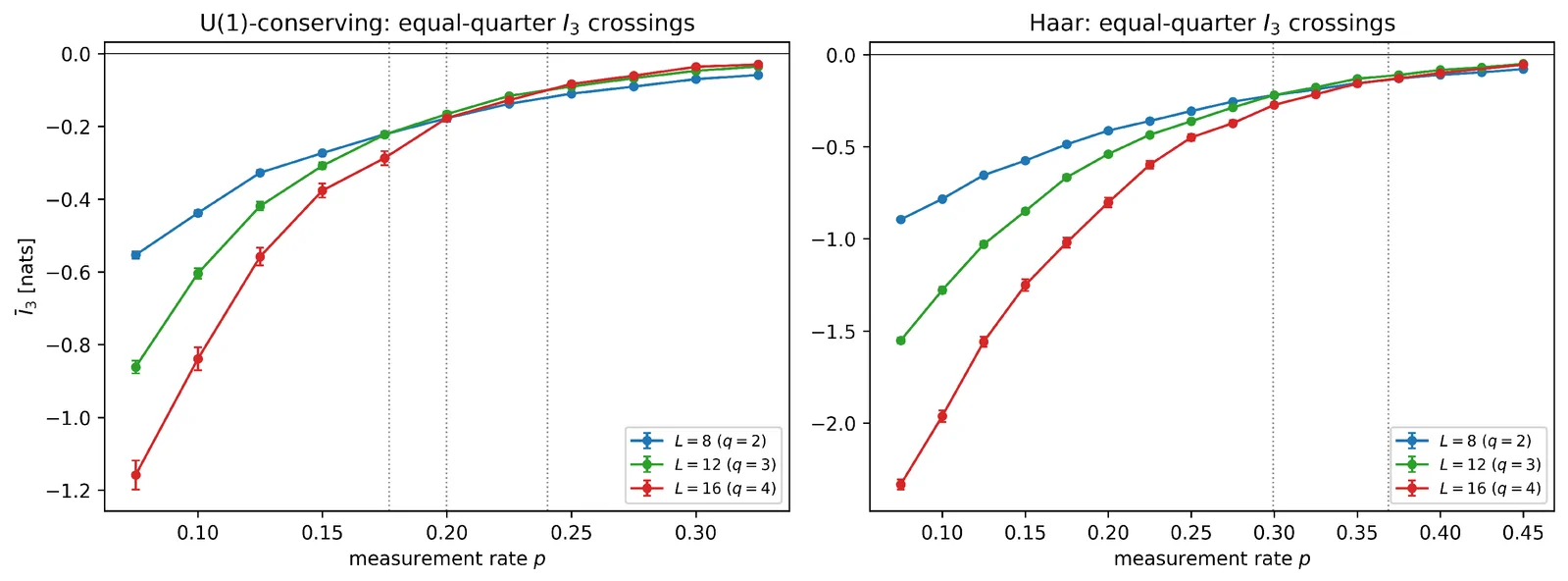
Tripartite mutual information $I_{3}$ versus measurement rate for equal-quarter system sizes L=8,12,16. Results are shown for the U(1)-conserving circuit (**left**) and the unconstrained Haar circuit (**right**). The dotted vertical lines mark representative pairwise crossings. Their remaining drift with system size is retained as a finite-size systematic rather than interpreted as statistical precision on the thermodynamic critical point.

As shown in Figure 4, the pairwise crossing locations drift toward larger *p* as the system size increases. We summarize the observed crossing regions by

(32) $$p_{\times }^{U(1)}≃0.21\pm 0.03,\;p_{\times }^{\mathrm{Haar}}≃0.34\pm 0.05,$$

where the quoted ranges span the finite-size drift and should not be read as thermodynamic critical-point error bars. The robust feature needed below is the ordering: within the same geometry and measurement protocol, the conserving reference crosses at a lower measurement probability than the unconstrained reference.

Agrawal et al. [7] likewise found a lower transition probability for a related U(1)-conserving monitored circuit than for its unconstrained counterpart. Their measurement schedule differs from ours, however, and the critical measurement probability is nonuniversal. We therefore do not convert their quoted probabilities by a fixed numerical factor or use such a conversion as quantitative validation of Equation (32). In the remainder of the paper the two endpoint circuits are used simply as internally consistent reference behaviors for the driven protocols.

## 4. Driven Symmetry-Breaking Protocols

We now make the mixing parameter of Section 3.2 explicitly time-dependent. The resulting circuit is a controlled nonstationary protocol: ϵ(t) is prescribed externally and selects a path between the U(1)-conserving and unconstrained Haar gate ensembles. It is not intended to emerge microscopically from accumulated errors. This distinction is important because the protocol itself fixes the direction of the gate interpolation. The informative observables are therefore the timing and structure of the many-body response.

**Figure 5:**
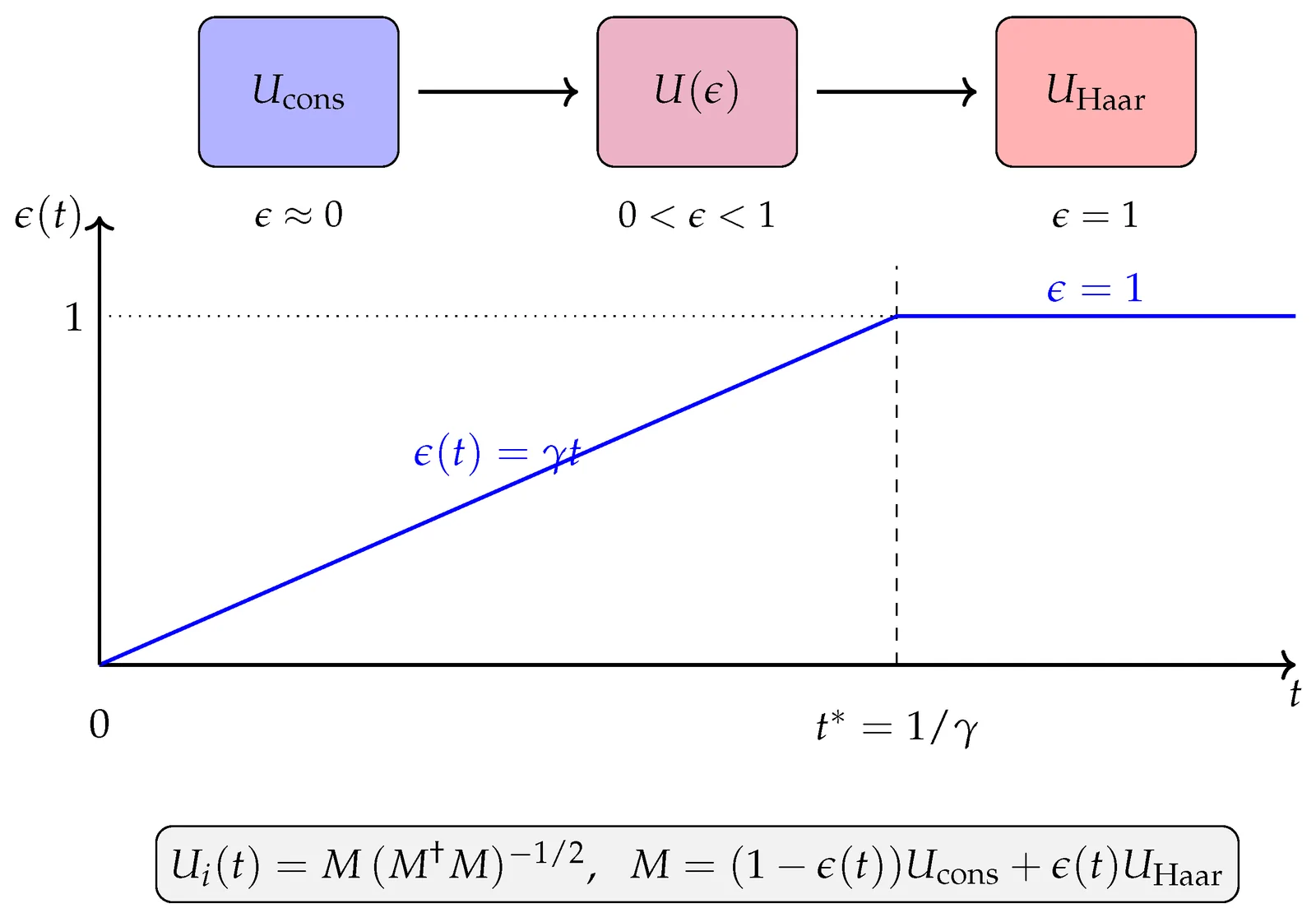
Externally imposed linear interpolation ϵ(t)=min(1,γt) between the two reference gate ensembles. The protocol endpoint time $t^{*}=1/\gamma$ is fixed by the drive and is distinct from response times extracted from the entanglement dynamics.

We use the same measurement protocol as in Section 3.4 and begin the pure-state calculations from the Néel product state, a standard half-filled initial condition for U(1)-symmetric monitored circuits [7,20]. We later repeat the comparison in the purification setting, where the physical system begins in the maximally mixed state. The externally imposed linear interpolation and its protocol endpoint are illustrated schematically in Figure 5.

### 4.1. Linear Symmetry-Breaking Ramp

At time step *t*, each two-site gate is obtained from the interpolating family of Section 3.2,

(33) $$U_{i}\left(t\right)=M_{i}\left(t\right)\;{\left[M_{i}{\left(t\right)}^{†}M_{i}\left(t\right)\right]}^{-1/2},\;M_{i}\left(t\right)=\left(1-\epsilon (t)\right)U_{\mathrm{cons}}+\epsilon \left(t\right)\;U_{\mathrm{Haar}},$$

with

(34) $$\epsilon_{\mathrm{lin}}\left(t\right)=\min\left(1,\gamma t\right).$$

The rate γ sets the externally imposed sweep timescale. At t=0 all gates are exactly conserving; at $t^{*}=1/\gamma$ the interpolation reaches the unconstrained endpoint. The system need not wait until $t^{*}$ to respond, and we extract its response times directly from the entropy dynamics below.

### 4.2. Exponential Symmetry-Breaking Ramp

To test sensitivity to the shape of the imposed drive, we also use

(35) $$\epsilon_{\exp}\left(t\right)=1-e^{-\gamma t}.$$

Unlike the linear ramp, this profile approaches ϵ=1 asymptotically. The two drives nevertheless share the same initial slope. For γt≪1,

(36) $$1-e^{-\gamma t}=\gamma t-\frac{1}{2}{\left(\gamma t\right)}^{2}+O\;\left({\left(\gamma t\right)}^{3}\right).$$

**Figure 6:**
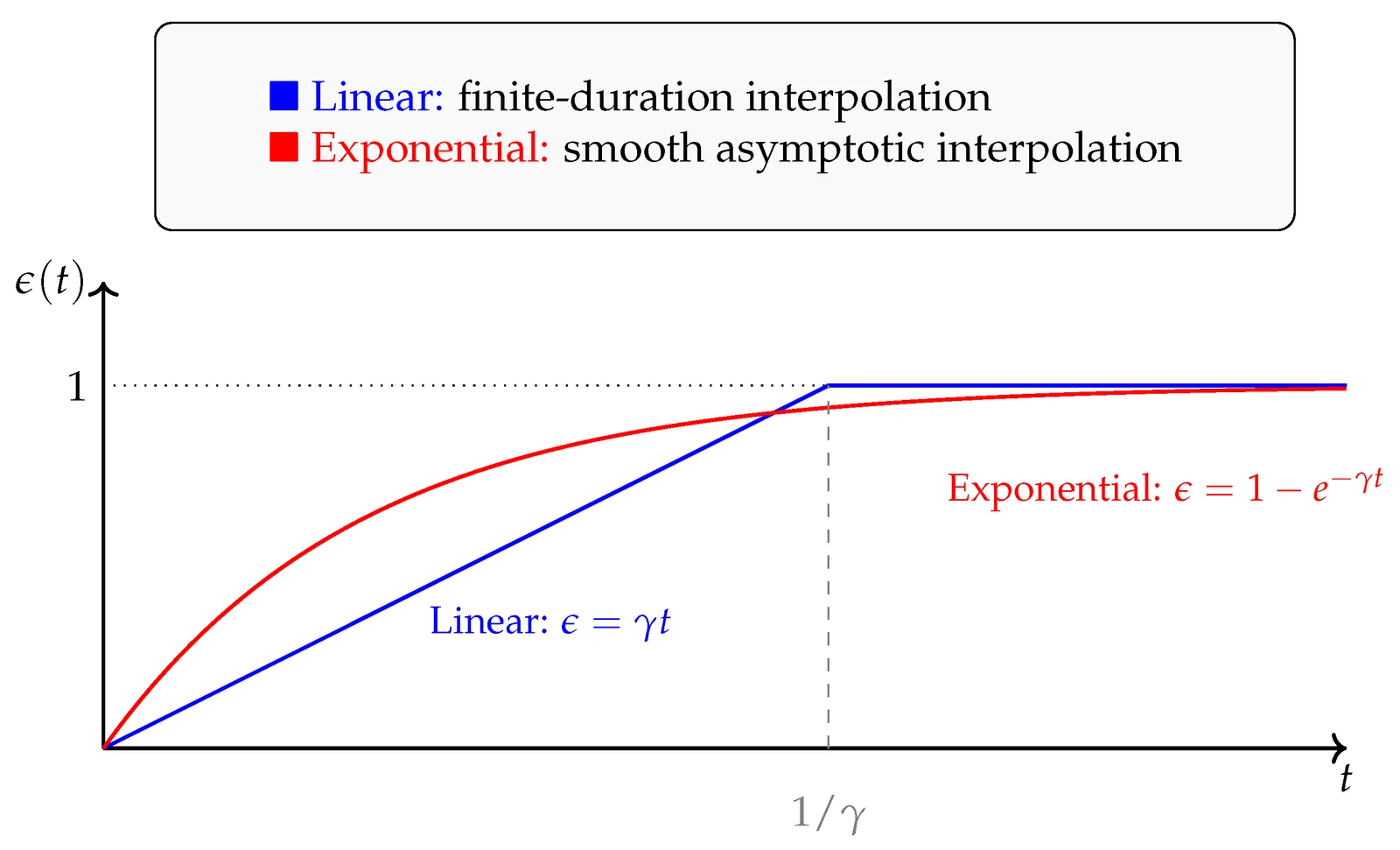
Comparison of the two externally prescribed symmetry-breaking profiles. They agree to leading order for γt≪1 but differ at later times: the linear protocol reaches ϵ=1 at 1/γ, whereas the exponential protocol approaches the same endpoint asymptotically.

Thus, the comparison probes two smooth protocols that agree at leading order at shallow depth but differ at later times. The exponential form is used here as a protocol choice, not as a microscopic model of leakage or another open-system process. The two prescribed symmetry-breaking profiles are compared schematically in Figure 6.

### 4.3. Numerical Results

We study the response to symmetry breaking using four related diagnostics. First, the late-time half-chain entropy shows where the driven circuit lies relative to the two reference behaviors at the end of the simulated time window. Second, the operational threshold times $t_{25}$, $t_{50}$, and $t_{75}$ characterize the timing and width of the transient response. Third, a constant-ϵ control separates the effect of an explicitly increasing drive from evolution under a weak fixed perturbation. Fourth, the residual entropy in the purification protocol tests whether a corresponding ramp crossover appears when the system begins in a maximally mixed state.

#### 4.3.1. Late-Time Half-Chain Entropy

We first measure the half-chain von Neumann entropy. For each pure-state trajectory, the system is divided into two equal halves, *A* and $\bar{A}$, and the reduced density matrix of *A* is

(37) $$\rho_{A}\left(t\right)={\mathrm{Tr}}_{\bar{A}}\left[|\psi (t)\rangle \langle \psi (t)|\right].$$

The half-chain entropy is then

(38) $$S_{A}\left(t\right)=-\mathrm{Tr}\left[\rho_{A}\left(t\right)\ln\rho_{A}\left(t\right)\right].$$

This quantity measures the entanglement between the two halves of the system. A larger value indicates greater bipartite entanglement, whereas a smaller value indicates stronger suppression of entanglement by the measurements.

To characterize the behavior near the end of each simulation, we average the trajectory-averaged entropy over the final 20 circuit layers:

(39) $${\bar{S}}_{\mathrm{late}}=\frac{1}{20}\sum_{t=T-19}^{T}{\langle S_{A}\left(t\right)\rangle }_{\mathrm{traj}}.$$

We calculate this quantity at L=12 and T=200 for γ∈{0,0.01,0.02,0.05,0.1}. By comparing ${\bar{S}}_{\mathrm{late}}$ with the values obtained from the U(1)-conserving and Haar reference circuits, we determine which reference behavior the ramped circuit more closely resembles within the simulated time window.

**Figure 7:**
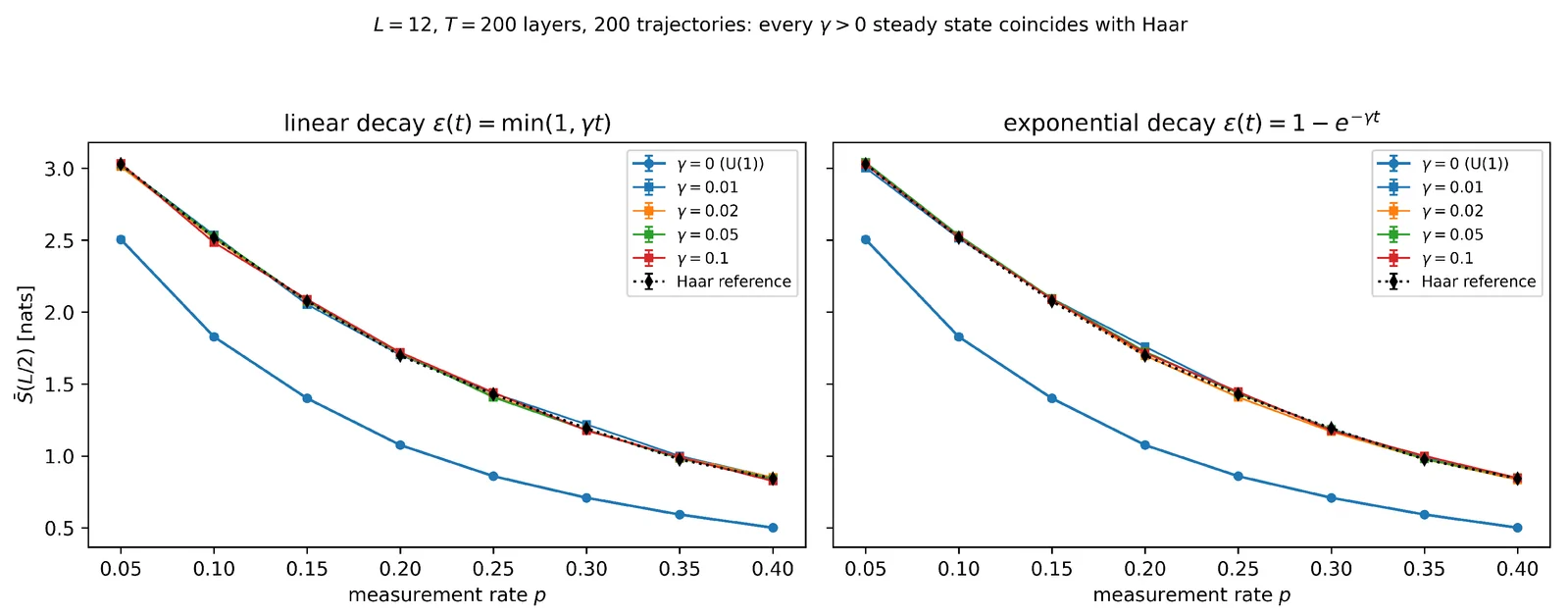
Late-time half-chain entropy versus measurement rate at L=12 for the linear ramp (**left**) and exponential ramp (**right**). The entropy is averaged over the final 20 layers of simulations with T=200 and compared with the γ=0 [U(1)-conserving] and Haar reference circuits. For the simulated nonzero ramp rates, the entropy moves toward the Haar reference. The largest remaining finite-time deviation occurs for the slowest exponential ramp, γ=0.01.

Figure 7 shows that most of the simulated nonzero-rate curves are statistically consistent with the Haar reference within our uncertainties. The main exception is the slowest exponential ramp, γ=0.01, for which the difference reaches approximately 3σ at individual measurement rates.

This remaining difference is consistent with an incomplete crossover during the finite simulation. For the ramp γ=0.01,

(40) $$\epsilon \left(200\right)=1-e^{-0.01\times 200}\approx 0.865,$$

so the gate interpolation has not yet reached the unconstrained endpoint by the final circuit layer. The results therefore show a finite-time movement toward the unconstrained reference behavior. They do not determine the asymptotic behavior of a circuit with a fixed, arbitrarily weak symmetry-breaking strength.

#### 4.3.2. Operational Crossover Times and Crossover Width

Because the ramp protocol explicitly moves the gate ensemble toward the Haar endpoint, we do not assign special physical significance to a single midpoint. Instead, we characterize the finite-time response using three operational thresholds. Let $S_{U(1)}^{\mathrm{ref}}$ and $S_{\mathrm{Haar}}^{\mathrm{ref}}$ denote the late-time entropies of the two reference circuits at the same measurement rate, and define

(41) $$S_{q}=S_{U(1)}^{\mathrm{ref}}+q\left(S_{\mathrm{Haar}}^{\mathrm{ref}}-S_{U(1)}^{\mathrm{ref}}\right),\;q\in \left\{0.25,0.50,0.75\right\}.$$

We define $t_{q}$ as the first recorded circuit layer at which the trajectory-averaged entropy reaches $S_{q}$. Thus the midpoint used in the original analysis is simply $t_{50}$. No temporal smoothing or interpolation is used for the primary extraction.

For γ=0.01,0.02,0.05, and 0.1, respectively, the extracted threshold times are

(42) $$\begin{array}{cccc} \left(t_{25},t_{50},t_{75}\right) & =(20,36,41),(15,20,24),(10,11,12),(7,8,9)\; & \; & \mathrm{at}\;p=0.1, \end{array}$$

(43) $$\begin{array}{cccc} \left(t_{25},t_{50},t_{75}\right) & =(29,41,50),(15,21,25),(8,10,13),(6,7,10)\; & \; & \mathrm{at}\;p=0.2. \end{array}$$

For clarity, these values are also summarized in Table 1.

**Table 1:** Operational crossover times extracted from the archived time-series data underlying Figure 8. The crossover width is $\Delta t_{\mathrm{cross}}=t_{75}-t_{25}$.

*p*	γ	$t_{25}$	$t_{50}$	$t_{75}$	$\Delta t_{\mathrm{cross}}$
0.1	0.01	20	36	41	21
0.1	0.02	15	20	24	9
0.1	0.05	10	11	12	2
0.1	0.10	7	8	9	2
0.2	0.01	29	41	50	21
0.2	0.02	15	21	25	10
0.2	0.05	8	10	13	5
0.2	0.10	6	7	10	4

**Figure 8:**
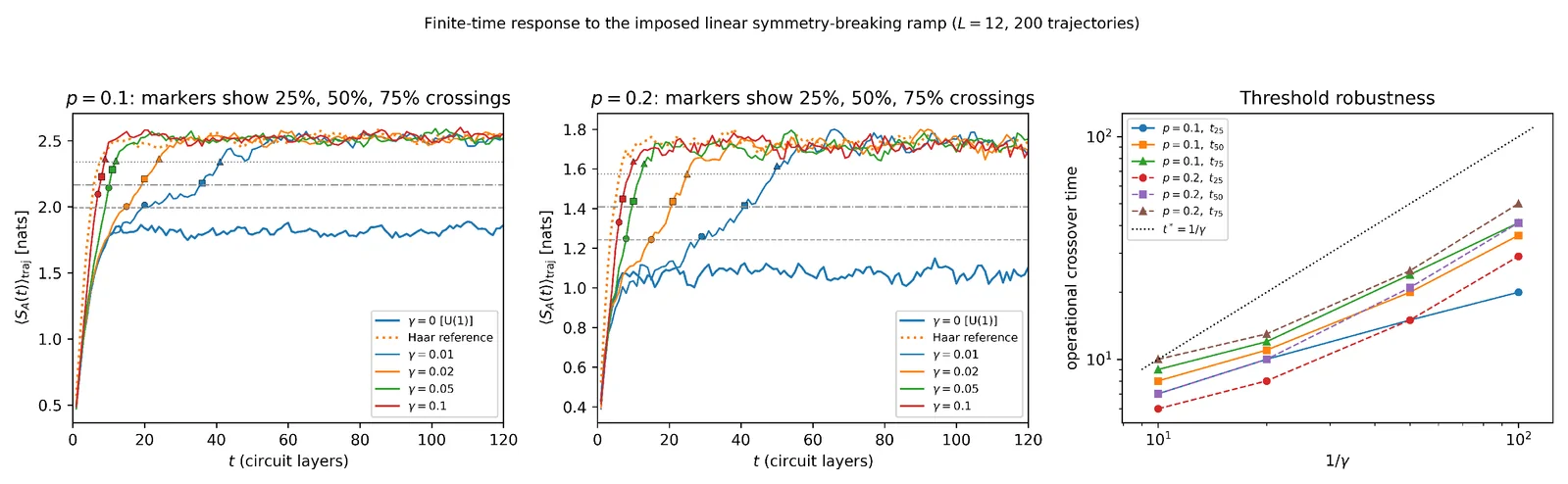
Finite-time response under the linear ramp for L=12 and 200 trajectories. **Left** and **center**: trajectory-averaged entropy at p=0.1 and p=0.2. The solid blue curve without threshold markers is the γ=0U(1)-conserving reference, whereas the blue γ=0.01 ramp curve carries threshold-crossing markers. Markers on each nonzero-γ curve indicate its first crossings of the 25%, 50%, and 75% levels between the late-time conserving and Haar reference entropies; the corresponding horizontal guide lines are shown in gray. **Right**: the extracted $t_{25}$, $t_{50}$, and $t_{75}$ versus 1/γ. The dotted line $t^{*}=1/\gamma$ is the endpoint time of the imposed linear protocol, not a fitted response law. No power-law fit is assumed.

All three thresholds shift systematically to later times as γ decreases. The conclusion that slower drives produce a later dynamical response therefore does not depend on selecting the 50% level. The temporal width

(44) $$\Delta t_{\mathrm{cross}}=t_{75}-t_{25}$$

is also larger for the slowest ramps within the range studied, indicating a broader transient response. We regard $t_{q}$ and $\Delta t_{\mathrm{cross}}$ as finite-time operational diagnostics rather than universal relaxation times.

The primary threshold extraction uses the unsmoothed trajectory-averaged time series. As a robustness check, we repeated it after centered moving averages with windows of 3, 5, and 7 circuit layers. Across the cases in Table 1, each extracted threshold changes by at most one circuit layer. The ordering with γ and the broadening of the slowest crossovers are therefore insensitive to moderate temporal averaging.

The endpoint time $t^{*}=1/\gamma$ of the linear drive remains useful as a protocol scale, but it should not be confused with a dynamical response time. The extracted thresholds occur before or, for the fastest p=0.2 case at the 75% level, at this endpoint. More importantly, the four available ramp rates do not provide enough dynamic range to identify a unique asymptotic functional form for $t_{q}\left(\gamma \right)$. Smooth alternatives, including power-law and forms containing a term proportional to 1/γ, can describe these few points comparably well. We therefore do not assign a universal crossover exponent.

#### 4.3.3. Matrix-Product-State Validation and Larger-System Check

The exact-statevector calculations above are limited to L≤16. To test whether the rate-ordering of the operational crossover survives beyond that limit, we implemented an independent matrix-product-state (MPS) simulation of the same monitored circuit. The MPS is maintained in mixed-canonical form. Each two-site gate is applied locally and split by singular-value decomposition, with a maximum bond dimension χ and an SVD cutoff of $10^{-10}$; the discarded Schmidt weight is recorded at every truncation. The MPS calculation uses the same gate ensembles, polar interpolation, odd-bond–measurement–even-bond ordering, computational-basis Born-rule measurements, and Néel initial state as the exact-statevector code.

We first validated the implementation without truncation. For a seeded L=8, p=0.2, γ=0.05 trajectory evolved for 20 layers, the final MPS and exact statevector agree at machine precision: the fidelity differs from unity by approximately $4\times 10^{-16}$ and the maximum phase-aligned amplitude difference is $3.5\times 10^{-15}$. Half-chain entropies agree to approximately $10^{-14}$ in additional untruncated tests up to L=16. We then performed an ensemble benchmark at L=16, p=0.4, T=40, and 12 matched trajectory seeds. With χ=64, the largest difference between the trajectory-averaged exact and MPS entropy curves among the U(1), Haar, and two ramped cases is $3.6\times 10^{-5}$. Using the final 10 layers to define the two reference entropies, the extracted threshold times are identical in the two methods: $\left(t_{25},t_{50},t_{75}\right)=\left(10,20,26\right)$ for γ=0.02 and (2,12,21) for γ=0.05.

For the larger-system check, we choose p=0.4, where monitored entanglement is sufficiently weak for controlled bond-dimension truncation. Table 2 shows the threshold times at L=16,24,32. At every size, the slower ramp γ=0.02 has a later $t_{50}$ and $t_{75}$ than γ=0.05.

At L=32, increasing the bond dimension from χ=96 to 128 changes the trajectory-averaged entropy curve by at most $2.2\times 10^{-4}$ for γ=0.02 and $6.4\times 10^{-4}$ for γ=0.05. For the χ=128 ramp calculations, the largest cumulative discarded weight in any trajectory is $3.7\times 10^{-4}$. The threshold values are therefore converged well before χ=128 in this weak-entanglement regime.

The threshold times are not monotonic in *L* in this 12-trajectory finite-size check. We therefore do not use the MPS data for a thermodynamic extrapolation or to infer a universal exponent. Their role is narrower but important: the slower-ramp/later-response ordering found in the exact calculations persists at L=24 and 32 when the MPS truncation is explicitly controlled. Extending this analysis deeper into the entangled regime would require substantially larger bond dimensions and trajectory ensembles.

#### 4.3.4. Constant Weak Symmetry Breaking as a Finite-Time Control

**Figure 9:**
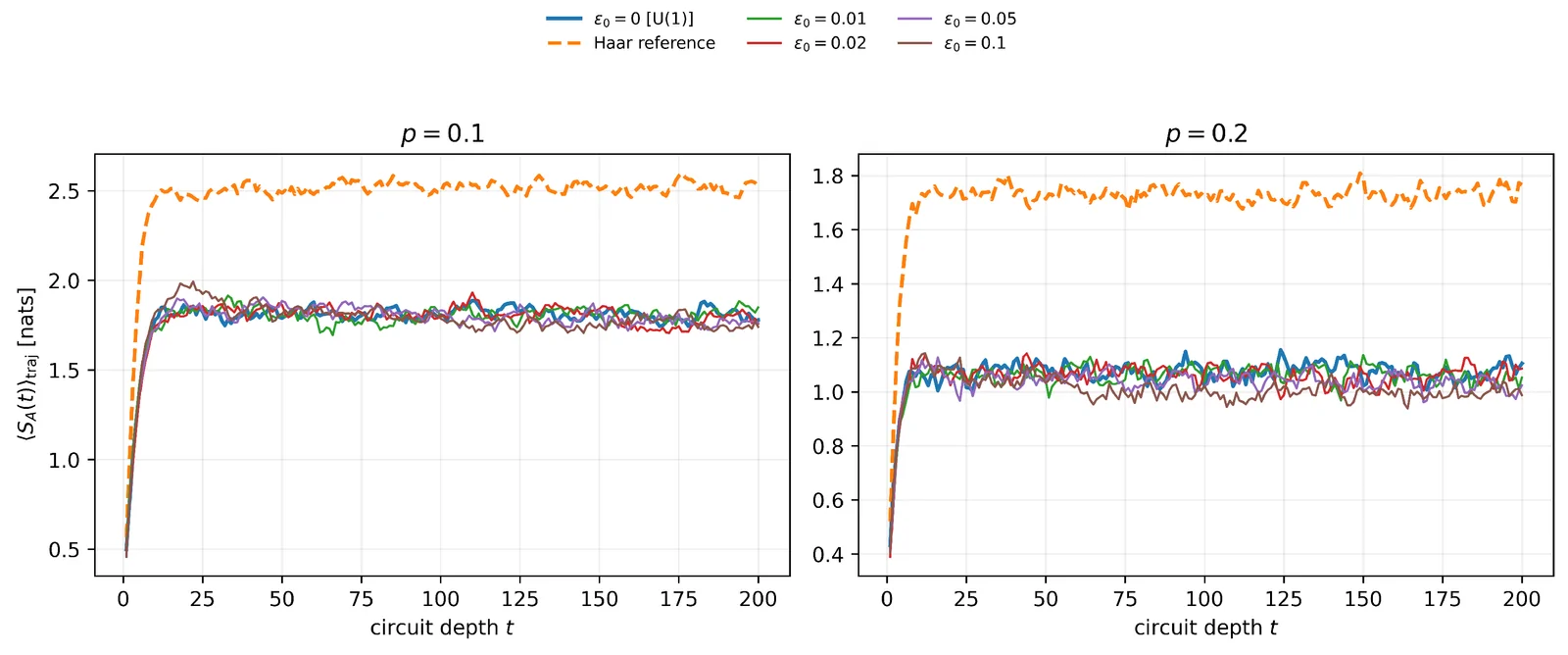
Finite-time control with a static symmetry-breaking strength $\epsilon \left(t\right)=\epsilon_{0}$ for L=12, T=200, and 200 trajectories. Results are shown at p=0.1 (**left**) and p=0.2 (**right**), together with the conserving ($\epsilon_{0}=0$) and Haar reference time series. For $\epsilon_{0}=0.01$–0.10, the static weak-perturbation curves remain close to the conserving reference throughout the simulated window and do not exhibit a sustained drift toward the Haar reference.

The ramp protocols above increase the gate-level symmetry-breaking strength with circuit depth. To test whether the observed response could instead arise simply from accumulating evolution under a weak but fixed perturbation, we perform a separate control in which

(45) $$\epsilon \left(t\right)=\epsilon_{0}$$

for the entire circuit. We use the same polar-interpolated gate family as in Equation (33), but hold the mixing parameter fixed at $\epsilon_{0}\in \left\{0.01,0.02,0.05,0.10\right\}$. We simulate L=12, T=200, and 200 trajectories at p=0.1 and p=0.2, matching the system size, time window, and trajectory count used for the linear-ramp time series. The resulting finite-time static-control trajectories are shown in Figure 9.

To quantify the late-time location of the static curves, define

(46) $$X_{\mathrm{late}}=\frac{{\bar{S}}_{\mathrm{late}}\left(\epsilon_{0}\right)-S_{U(1)}^{\mathrm{ref}}}{S_{\mathrm{Haar}}^{\mathrm{ref}}-S_{U(1)}^{\mathrm{ref}}},$$

where the reference values and ${\bar{S}}_{\mathrm{late}}$ are averages over the final 20 circuit layers. At p=0.1, the four values of $X_{\mathrm{late}}$ for $\epsilon_{0}=0.01,0.02,0.05,0.10$ are approximately 0.01,−0.04,−0.09,−0.12, respectively; at p=0.2 they are approximately −0.05,−0.01,−0.09,−0.12. Thus, all eight late-time static controls remain within about 12% of the conserving reference when measured in units of the full conserving-to-Haar entropy separation. The small negative values are finite-size and finite-sampling offsets relative to the chosen reference and are not interpreted as a distinct phase.

The contrast with Figure 8 is important. The driven ramps reach intermediate and ultimately large values of ϵ(t), whereas the static controls remain weak at every depth. Over the present 200-layer window, weak fixed symmetry breaking therefore does not reproduce the sustained crossover produced by the imposed ramps. This result does not establish that a constant nonzero $\epsilon_{0}$ is asymptotically irrelevant: a much longer-time and larger-system study would be required to determine whether an arbitrarily weak static perturbation eventually changes the long-time universality class.

#### 4.3.5. Purification Dynamics with the Maximally Mixed Initial State

The pure-state simulations above measure the spatial entanglement generated from a Néel product state. Although this is a standard diagnostic of a measurement-induced transition, it leaves open whether the observed symmetry-breaking crossover depends on that particular initial state. We therefore perform a complementary purification calculation beginning from a maximally mixed state.

This calculation asks whether the monitored circuit preserves or removes initially stored quantum information. If the information is preserved, the system remains entangled with an external reference system. If repeated measurements remove the information, the system decouples from the reference and becomes pure. Observing the same symmetry-breaking crossover in this diagnostic would therefore show that the effect is not specific to the generation of spatial entanglement from the Néel state.

We initialize the system *S* in the maximally mixed state

(47) $$\rho_{S}\left(0\right)=\frac{I}{2^{L}}.$$

To represent this mixed state within a pure-state calculation, we introduce an auxiliary reference system *R*. Each system qubit $S_{i}$ is initially maximally entangled with a corresponding reference qubit $R_{i}$:

(48) $$|\Psi_{SR}\left(0\right)\rangle =\overset{L}{\underset{i=1}{⨂}}\frac{{|0\rangle }_{S_{i}}{|0\rangle }_{R_{i}}+{|1\rangle }_{S_{i}}{|1\rangle }_{R_{i}}}{\sqrt{2}}.$$

Tracing out the reference gives

(49) $${\mathrm{Tr}}_{R}\left[|\Psi_{SR}\left(0\right)\rangle \langle \Psi_{SR}\left(0\right)|\right]=\frac{I}{2^{L}},$$

so this enlarged pure state reproduces the desired maximally mixed state of the physical system.

The circuit gates and measurements act only on *S*; no operation is applied directly to *R*. The reference therefore serves as a record of the quantum information initially stored in the system.

For each measurement trajectory, the conditioned joint state of *S* and *R* remains pure. We measure the residual purification entropy

(50) $$S_{\mathrm{pur}}\left(t\right)\equiv S\;\left(\rho_{S}\left(t\right)\right)=-\mathrm{Tr}\left[\rho_{S}\left(t\right)\ln\rho_{S}\left(t\right)\right],$$

where

(51) $$\rho_{S}\left(t\right)={\mathrm{Tr}}_{R}\left[|\Psi_{SR}\left(t\right)\rangle \langle \Psi_{SR}\left(t\right)|\right].$$

Because the joint state is pure,

(52) $$S\;\left(\rho_{S}\left(t\right)\right)=S\;\left(\rho_{R}\left(t\right)\right),$$

and this entropy measures the entanglement remaining between the system and the reference.

Initially,

(53) $$S_{\mathrm{pur}}\left(0\right)=L\ln2,$$

because the system and reference are maximally entangled. If $S_{\mathrm{pur}}\left(t\right)$ remains nonzero, the monitored circuit has retained some of the initially encoded quantum information. If $S_{\mathrm{pur}}\left(t\right)\to 0$, the system has decoupled from the reference and has been purified by the measurements.

We simulate this purification protocol for L=6 and L=8 up to a circuit depth of T=80. For each measurement rate, we calculate the trajectory-averaged residual entropy

(54) $${\bar{S}}_{\mathrm{pur}}\left(T\right)={\langle S_{\mathrm{pur}}\left(T\right)\rangle }_{\mathrm{traj}}.$$

**Figure 10:**
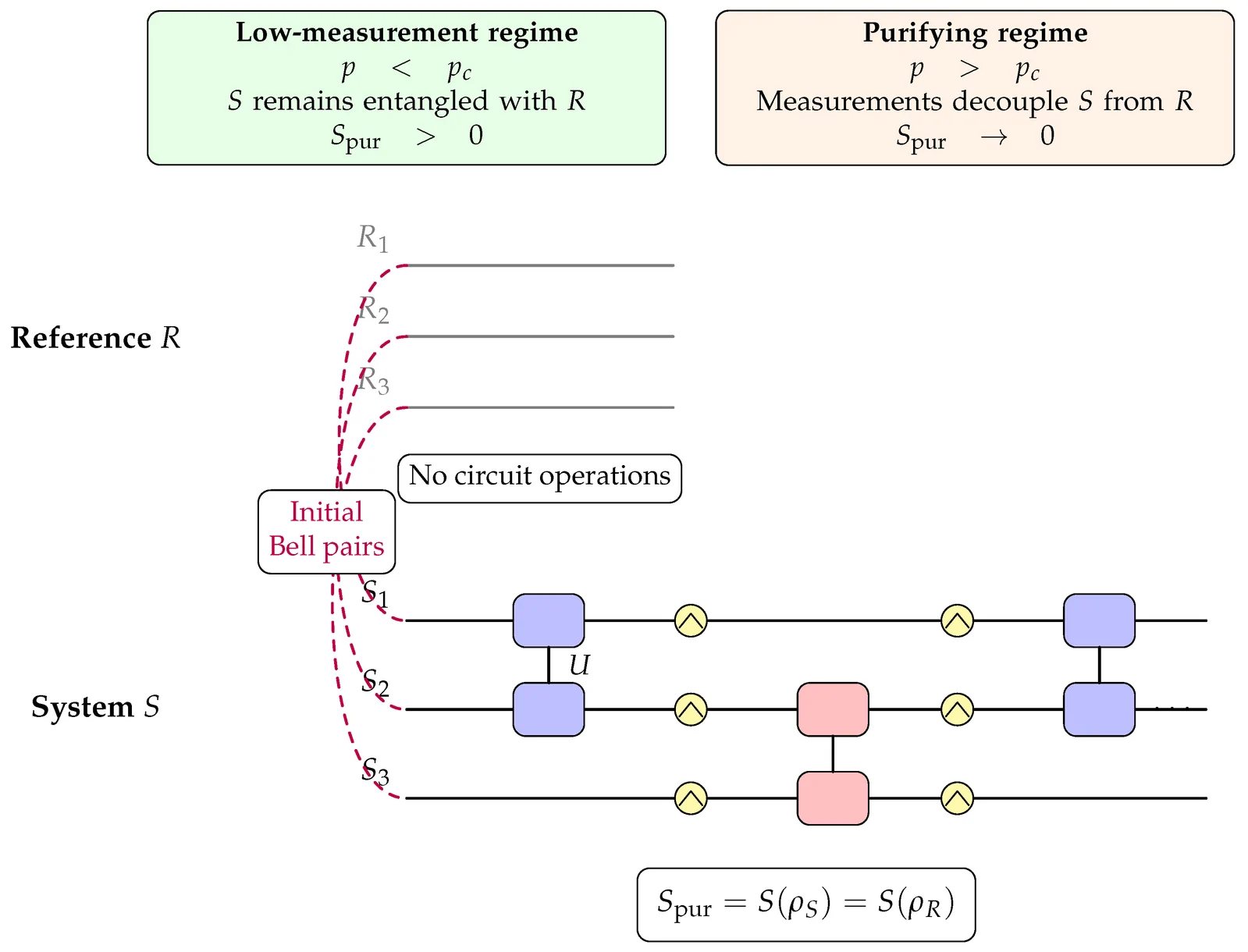
Purification dynamics in the reference-system construction. The maximally mixed state of the physical system *S* is represented as part of an initially pure entangled state of *S* and *R*. Gates and measurements act only on *S*; no circuit operation is applied directly to *R*. The residual entropy $S_{\mathrm{pur}}=S\left(\rho_{S}\right)=S\left(\rho_{R}\right)$ measures the system–reference entanglement remaining along each trajectory. In the purifying regime, measurements progressively decouple *S* from *R*, causing $S_{\mathrm{pur}}$ to approach zero.

We compare the linear and exponential symmetry-breaking ramps at γ∈{0.02,0.05,0.1} with the U(1)-conserving and Haar reference circuits. The system–reference purification construction is illustrated in Figure 10.

**Figure 11:**
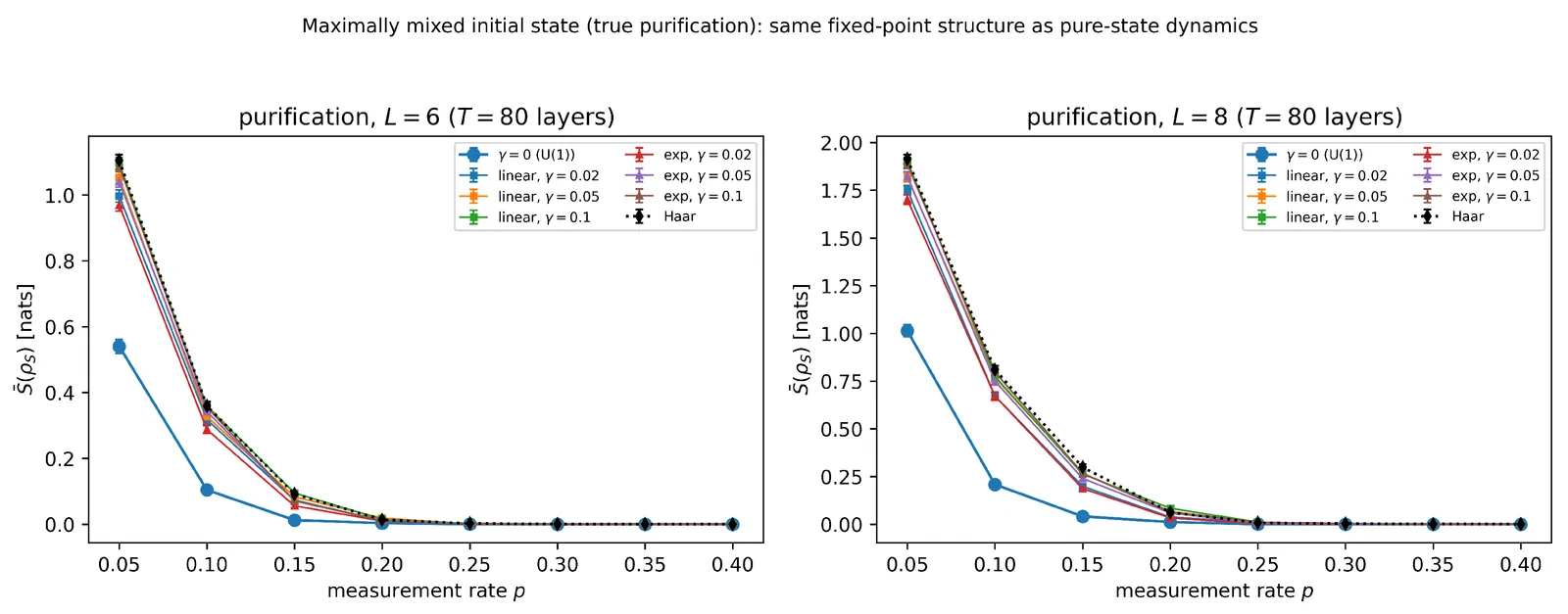
Trajectory-averaged residual purification entropy ${\bar{S}}_{\mathrm{pur}}\left(T\right)$ versus measurement rate for L=6 (**left**) and L=8 (**right**) after T=80 circuit layers. Results are shown for the linear and exponential symmetry-breaking ramps with γ∈{0.02,0.05,0.1} and for the U(1)-conserving and Haar reference circuits.

Figure 11 shows how the residual purification entropy depends on the measurement rate. At large *p*, the residual entropy approaches zero for every simulated gate ensemble. Frequent measurements therefore decouple the system from the reference and purify the system within the simulated time window.

At smaller *p*, the residual entropy remains nonzero, showing that some system–reference entanglement survives. The U(1)-conserving circuit retains less system–reference entanglement than the nonzero-rate ramped circuits, while increasing the ramp rate moves the results toward the Haar reference.

Quantitatively, for both symmetry-breaking profiles, the mean deviations from the Haar reference are approximately 1.5σ or less when γ≥0.05. At the slowest simulated rate, γ=0.02, deviations of up to approximately 3σ remain at some measurement rates. These larger deviations are consistent with the crossover being less complete within the finite simulation time.

The purification results therefore support the main conclusion obtained from the pure-state simulations: over the ramp rates and evolution times studied, progressively increasing symmetry breaking produces a crossover from the U(1)-conserving behavior toward the unconstrained Haar behavior. Because the same qualitative crossover occurs when the system begins in a maximally mixed state, it is not specific to the Néel initial state.

## 5. Discussion and Implications

### 5.1. What the Simulations Establish

The endpoint calculations provide two finite-size reference behaviors under the same circuit geometry and measurement schedule. The equal-quarter $I_{3}$ crossings occur near $p_{\times }^{U(1)}≃0.21$ and $p_{\times }^{\mathrm{Haar}}≃0.34$, with appreciable residual drift across L=8,12,16. These numbers are therefore reference crossing locations, not thermodynamic-limit critical-point determinations.

Under the externally imposed linear and exponential drives, the entanglement dynamics move away from the conserving reference and toward the unconstrained reference. Since that direction is built into the protocol, the more informative result is the rate-dependent transient response. For the linear drive, $t_{25}$, $t_{50}$, and $t_{75}$ all move to later depths as γ decreases, and this conclusion is unchanged by moderate temporal averaging. The interval $t_{75}-t_{25}$ provides an additional measure of crossover width and is largest for the slowest ramps studied.

The independent MPS calculation provides a controlled larger-system check of this rate ordering. After reproducing the exact-statevector thresholds at L=16, it preserves the slower-ramp/later-response ordering at L=24 and 32 for p=0.4, with the L=32 thresholds unchanged between χ=64,96, and 128. Because the finite-size thresholds are not monotonic in *L*, we interpret this as a robustness check rather than a thermodynamic extrapolation.

The constant-ϵ control gives a complementary finite-time result. For $\epsilon_{0}=0.01$–0.10 at L=12 and T=200, the entropy remains close to the conserving reference at both p=0.1 and 0.2 and exhibits no sustained motion toward the Haar reference. Thus, the driven response is not reproduced simply by accumulating the same circuit depth under a weak fixed symmetry-breaking gate ensemble. This statement is intentionally limited to the simulated window and does not resolve the asymptotic relevance of static symmetry breaking.

Purification simulations beginning from the maximally mixed state exhibit a corresponding crossover in the residual system-reference entropy. This gives an independent information-theoretic diagnostic and shows that the observed finite-time response is not specific to entanglement growth from the Néel product state.

### 5.2. Finite-Size, Scaling, and Protocol Limitations

Exact statevector evolution avoids bond-dimension truncation and preserves the simulated trajectory dynamics exactly up to floating-point arithmetic, but it limits the primary calculations to L≤16. The MPS check in Section 4.3.3 extends the crossover analysis to L=24 and 32 at p=0.4 and verifies convergence up to χ=128. This larger-system check reduces, but does not eliminate, the finite-size limitation. The thresholds are not monotonic in *L*, only 12 trajectories are used in the MPS pilot, and the calculation is deliberately restricted to a weak-entanglement regime where truncation can be controlled. We therefore make no thermodynamic-limit claim for either the crossing locations or the functional dependence of $t_{q}$ on γ. Extending the same controlled convergence tests to more strongly entangled regimes and larger trajectory ensembles would require substantially higher bond dimensions and additional computation.

The limited ramp-rate range is a second constraint. With four nonzero values of γ, several smooth functions describe the threshold times well, so the data do not select a unique power law and do not support a universal crossover exponent. The threshold analysis instead isolates the robust statement that the entire 25–75% response window shifts later as the drive is slowed.

A third limitation concerns the distinction between finite-time and asymptotic response. We added a constant-ϵ control with $\epsilon_{0}=0.01$–0.10 and find no sustained drift toward the Haar reference within T=200 at L=12 for p=0.1 or 0.2. This separates the ramp-driven crossover from simple accumulation under a weak fixed gate ensemble over the same time window. It does not, however, determine whether a constant perturbation

(55) $$\epsilon \left(t\right)=\epsilon_{0},\;0<\epsilon_{0}\ll 1,$$

is ultimately relevant at much longer times or in the thermodynamic limit. That question requires larger systems and substantially longer evolutions. Likewise, additional product states, such as domain-wall or random fixed-charge-sector states, would help separate initial-state-dependent early dynamics from more robust late-time response.

Finally, ϵ(t) controls a coherent unitary gate ensemble. It does not prescribe a decay law for the charge observable and is not an open-system noise strength. Once the gates cease to conserve charge, the evolution of charge-dependent observables depends on the state, measurement outcomes, gate realizations, and system size.

### 5.3. Outlook for Hardware and Trainable Quantum Circuits

For quantum hardware, the present results should be interpreted as a controlled response study rather than a microscopic noise model. Physical amplitude damping, dephasing, and leakage act through non-unitary channels, and transmon leakage can populate levels outside the computational subspace. Our two-level unitary interpolation captures none of these processes microscopically. A direct hardware extension would replace the prescribed ϵ(t) ramp with calibrated quantum channels or a multilevel transmon model and then ask whether analogous operational threshold times can be identified.

Symmetry also plays an important role in variational quantum algorithms and quantum machine learning, where symmetry-preserving ansatzes can encode an inductive bias [21,22]. The present random monitored circuits, however, contain no trainable parameters, loss optimization, gradients, or generalization test. They therefore do not imply results about optimization landscapes, trainability, or barren plateaus. A meaningful extension would introduce a trainable symmetry-preserving ansatz, control the degree of symmetry breaking during optimization, and test whether any finite-time or finite-depth crossover correlates with expressivity, gradient statistics, optimization performance, or generalization. In this sense, the QML connection is a concrete direction for future work rather than a result of the current simulations.

## 6. Conclusions

We have studied the finite-time response of a monitored one-dimensional random circuit to externally prescribed U(1)-symmetry-breaking drives. Exact statevector simulations provide two distinct finite-size reference behaviors: the equal-quarter tripartite-mutual-information crossings lie near $p_{\times }^{U(1)}≃0.21$ for the conserving circuit and $p_{\times }^{\mathrm{Haar}}≃0.34$ for the unconstrained Haar circuit. Because these crossings drift across the accessible sizes, we use them as internal reference estimates rather than thermodynamic critical-point determinations.

For the linear and exponential drives, slower variation of the gate ensemble produces a later entanglement response. The linear-ramp time series make this statement robust to the operational definition of crossover. The 25%, 50%, and 75% threshold times all move later as γ decreases, while moderate 3-, 5-, and 7-layer moving averages change individual thresholds by at most one layer. The corresponding 25–75% interval also shows that the slowest ramps have the broadest transient response within the range studied. With only four nonzero ramp rates and small exact-statevector systems, however, the data do not determine a unique asymptotic scaling function and we do not assign a universal crossover exponent.

Purification calculations beginning from the maximally mixed state show a parallel crossover in the residual system-reference entropy. This independent diagnostic demonstrates that the finite-time response is not specific to the Néel initial state.

An independently implemented MPS calculation provides a larger-system consistency check. It reproduces the exact-statevector threshold extraction at L=16 and, with controlled bond-dimension convergence, preserves the slower-ramp/later-response ordering at L=24 and 32 for p=0.4. The finite-size threshold values are not monotonic in *L*, so this result supports the robustness of the ordering rather than a thermodynamic extrapolation.

A complementary static control sharpens the scope of this conclusion. For fixed $\epsilon_{0}=0.01$–0.10, L=12, and T=200, the entropy remains close to the conserving reference at both p=0.1 and 0.2 and shows no sustained crossover toward the Haar reference. The driven crossover is therefore not reproduced simply by evolving for the same duration under a weak fixed perturbation. The asymptotic fate of arbitrarily weak static symmetry breaking nevertheless remains unresolved.

The model is deliberately limited in scope. ϵ(t) is a coherent gate-ensemble control parameter rather than a physical decoherence or leakage probability. Larger MPS studies in more strongly entangled regimes, longer constant-ϵ controls, additional charge-sector initial states, and explicit open-system or multilevel noise models are therefore natural next steps. For trainable quantum circuits, a separate study would be needed to connect symmetry breaking to optimization landscapes, trainability, or generalization.

## Figures and Tables

**Table 2:** MPS larger-system check at p=0.4, T=40, and 12 trajectories. The L=16 and L=24 rows use χ=64. At L=32, the thresholds are unchanged for χ=64,96, and 128, demonstrating bond-dimension convergence of the operational crossover times.

*L*	χ	γ	$\left(t_{25},t_{50},t_{75}\right)$	Comment
16	64	0.02	(10,20,26)	exact-MPS benchmark
16	64	0.05	(2,12,21)	exact-MPS benchmark
24	64	0.02	(21,24,30)	larger-system check
24	64	0.05	(10,12,14)	larger-system check
32	64–128	0.02	(19,20,21)	converged in χ
32	64–128	0.05	(6,10,11)	converged in χ

## Data Availability

The numerical data underlying the figures and reported results, along with the exact-statevector and MPS simulation code, analysis scripts, and figure-reproduction code, are provided in the Supplementary Materials. Within the supplementary archive, these materials are organized under the data/, code/, analysis/, and mps/ directories.

## Supplementary Materials

The following supporting information can be downloaded at https://www.mdpi.com/article/10.3390/e28101120/s1: **Supplementary Code**: exact-statevector and MPS simulation code, analysis and threshold-extraction scripts, robustness-check scripts, the constant-ϵ control script, and the figure-reproduction code; **Supplementary Data**: numerical data underlying the manuscript figures and reported results, including the 25%, 50%, and 75% threshold extraction, moving-average robustness check, MPS validation data, and constant-ϵ control data. The supplementary archive also contains the manuscript figure files and README documentation. From the top level of the extracted archive, the documented command python reproduce_figures.py regenerates every data-driven manuscript figure.
